# Nonlinear Rayleigh wave propagation in thermoelastic media in dual-phase-lag

**DOI:** 10.1038/s41598-022-25680-7

**Published:** 2022-12-08

**Authors:** A. A. Youssef, N. K. Amein, N. S. Abdelrahman, M. S. Abou-Dina, A. F. Ghaleb

**Affiliations:** 1 Department of Mathematics, Faculty of Science, Suez Canal University, Ismailia, Egypt; 2 Department of Mathematics, Faculty of Science, Cairo University, Giza, 12613 Egypt

**Keywords:** Engineering, Materials science

## Abstract

A model of generalized thermoelasticity within dual-phase-lag is used to investigate nonlinear Rayleigh wave propagation in a half-space of a transversely isotropic elastic material. It is assumed that the coefficient of heat conduction is temperature-dependent, a fact that plays an important role in the coupling behaviour analysis of thermoelastic and piezo-thermoelastic solids. Taking such a dependence into account becomes a necessity at higher temperatures and in nano-structures, when the material properties can no longer be considered as constants. Normal mode analysis is applied to find a particular solution to the problem under consideration. A concrete case is solved under prescribed boundary conditions and tentative values of the different material coefficients. The results are discussed to reveal the effect of temperature dependence of the heat conduction coefficient, as well as the thermal relaxation times, on nonlinear Rayleigh wave propagation. All quantities of practical interest are illustrated in two-and three-dimensional plots. The presented results may be useful in the detection of the second harmonic amplitudes through measurements related to the propagating heat wave.

## Introduction

The use of wave propagation has many applications in Engineering and in Geophysics, for non-destructive testing of materials and for exploration purposes. Nonlinear Rayleigh waves are used for the characterization of damage due to plastic deformations in materials, especially in high temperature alloys. Hermann et al.^1^ developed a technique for measuring the second order harmonic amplitude of a Rayleigh surface wave propagating in metallic specimens and discussed its uses to assess the damage in nickel-base high temperature alloy specimens, and the evolution of material nonlinearity under various loading conditions. Shui et al.^2^ developed a new technique for measuring the acoustic nonlinearity of materials using Rayleigh waves. Walker et al.^3^ investigated the effect of plastic deformation on nonlinear Rayleigh surface waves and the possibility of using this kind of waves for the study of fatigue in structures. Doerr et al.^4^ studied the propagation of nonlinear Rayleigh waves in some types of stainless steels and its uses in the reduction of corrosion in these materials. Ding et al.^5^ investigated the mechanism of nonlinear Rayleigh waves for the detection of surface micro-cracks. Masurkar et al.^6^ presented a nondestructive testing model for the evaluation of dislocation-induced material nonlinearity in rails using an amplitude-based nonlinear parameter of Rayleigh wave propagation.

The theory of extended thermodynamics is appropriate for the study of wave propagation in materials of complex structure, as it yields finite speeds of propagation of heat and allows for the retardation effects and the energy dissipation associated with thermal relaxation to be explained. The importance of generalized thermoelasticity for the description of wave phenomena in continuum models of thermoelasticity appears in the existing extensive literature on the subject. We only cite here a few which are concerned with wave propagation in thermoelastic media under different theories, and with one, two or three thermal phase lags^7^-^19^. These are concerned only with linear phenomena. Other work was devoted to more general, nonlinear models involving electro-thermoelastic interactions^20,21^. The study of the nonlinear Rayleigh wave propagation has been considered mainly for nondestructive testing and for the evaluation of higher order material moduli.

The present work investigates nonlinear Rayleigh wave propagation in a half-space of a transversely isotropic thermoelastic material within dual-phase-lag theory. Nonlinearity here is conditioned by the dependence of the heat conduction coefficients on temperature. A small parameter is introduced, related to the small variations of temperature as measured from a reference temperature. It is well-known that temperature dependence of the material coefficients plays an important role in the understanding of thermoelastic and piezo-thermoelastic couplings in solids. In particular, temperature-dependent thermal conductivity is an important factor in many engineering problems involving thermo-mechanical analysis. Numerous experimental investigations have indicated that such a temperature dependence becomes necessary for an efficient description of the continuum, especially at higher temperatures and in nano-structures (C.f.^22^-^30^). When the nature of dependence on temperature is linear, as presently considered, then the coefficient of linearity appears only starting from the second order of approximation when the surface wave solution is expanded in a series in terms of a Poincaré small parameter. In other words, this coefficient is related to the first sub-harmonic solution. More generally, if the thermal conductivity is expanded in a power series in temperature, then the successive coefficients of this expansion will appear in the successive higher approximations and will be related to higher subharmonics. It is the purpose of the present work to propose a method for the evaluation of the linearity coefficient in the function of thermal conductivity, by assessing the response of the thermoelastic medium to Rayleigh wave propagation. When the model under consideration allows for heat to propagate as a wave, then one can use the dynamical thermal measurements as a tool for evaluating the above mentioned material coefficient.

The governing equations are presented in the form of a system of first order partial differential equations, which is advantageous for numerical and for other techniques as well. An approximate solution is obtained using the normal modes technique in the first two orders of approximation only. The results are discussed in detail, more specifically the influence of temperature dependence of the heat conduction coefficient, and the thermal relaxation times on wave propagation. The quantities of practical interest are represented graphically in two-and three-dimensional plots. The presented results may be useful in the experimental detection of the second harmonic amplitudes through measurements related to the heat wave propagation.

## Problem formulation

### Basic equations

A first order, two-dimensional system of nonlinear partial differential equations of plane strain thermoelasticity is presented within the frame of extended thermodynamics and dual-phase-lag theory. The system is composed of the equations of classical thermoelasticity for the balance of momentum and energy, complemented with the evolution equation for heat flux. This system is thermodynamically admissible and involves nonlinearity caused by the dependence of the coefficient of heat conduction on temperature.

**Figure 1:**
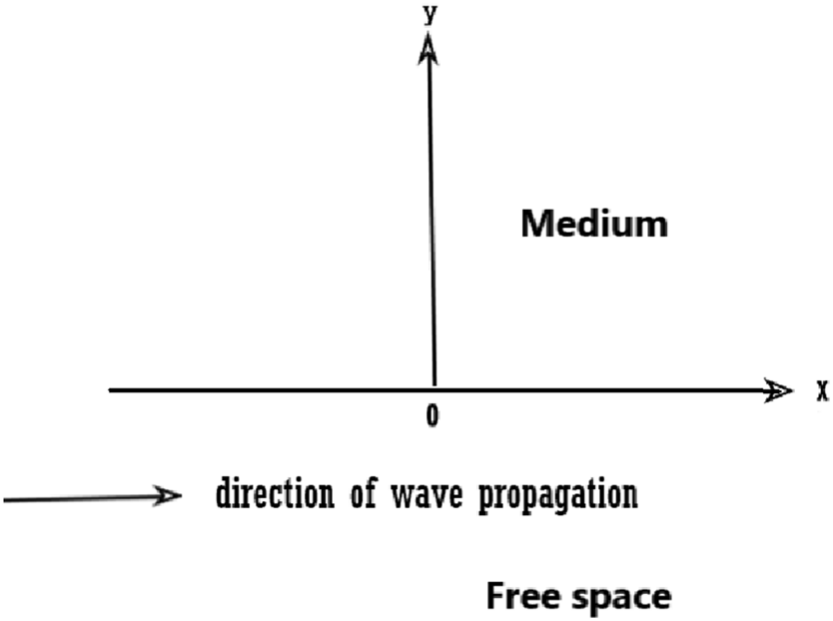
Geometry of the problem.

The theory of Extended Thermodynamics in the present context assumes that heat flux is a thermodynamical variable that is independent of temperature. It has its own evolution equation, called the Cattaneo-Vernotte equation, that replaces the classical Fourier law for heat conduction. This evolution equation involves a thermal relaxation time ($\tau _q$) related to the heat flux. A development of this theory, under the name of dual-phase-lag theory (DPL), introduced a second relaxation time ($\tau _{\theta }$) associated to temperature. The starting point for this theory is the relation between heat flux and temperature, which in simplified form reads (Cf.^19^):

$$\begin{aligned} \varvec{q}(\varvec{r},t + \tau _q) = - K \varvec{\nabla } [ \theta (\varvec{r},t + \tau _{\theta })]. \end{aligned}$$

A development of this last relation by Taylor’s series yields the required evolution equation for the heat flux in DPL. Setting $\tau _{\theta }=0$ gives the theory with one relaxation time.

The problem under consideration is that of nonlinear Rayleigh wave propagation in a half-space of a transversely isotropic thermoelastic material. It will be described in a system of orthogonal Cartesian coordinates (*x*, *y*, *z*) with origin *O* placed on the boundary of the half-space, the *y*-coordinate being directed into the depth of the material, as shown in Fig. 1.

Let $u_x,u_y$ denote the mechanical displacement components, $v_x,v_y-$ the corresponding velocity components, $\sigma _{xx}, \sigma _{yy}, \sigma _{xy}-$ the identically non-vanishing in-plane stress components, $\theta -$ the temperature as measured from a reference temperature $\theta _0$ and $q_x, q_y-$ the heat flux components.

In the absence of body forces and heat sources, the equations of plane thermoelasticity for a transversely isotropic material within the theory of extended thermodynamics to be considered read^19^:

1. Equations of motion:
  1 $$\begin{aligned}{} & {} \rho \frac{\partial v_x}{\partial t} - \frac{\partial \sigma _{xx}}{\partial x} - \frac{\partial \sigma _{xy}}{\partial y} = 0, \end{aligned}$$
  2 $$\begin{aligned}{} & {} \rho \frac{\partial v_y}{\partial t} - \frac{\partial \sigma _{xy}}{\partial x} - \frac{\partial \sigma _{yy}}{\partial y} = 0, \end{aligned}$$
2. Equation of heat conduction:
  3 $$\begin{aligned} \theta _0 \gamma \left( \frac{\partial v_x}{\partial x} + \frac{\partial v_y}{\partial y} \right) + \rho C_e \frac{\partial \theta }{\partial t} + \frac{\partial q_x}{\partial x} + \frac{\partial q_y}{\partial y} = 0, \end{aligned}$$
3. Evolution laws for the heat flux components (which replace the classical Fourier law for heat conduction):
  4 $$\begin{aligned}{} & {} \tau _q \frac{\partial q_x}{\partial t} + q_x + K_{11}(\theta ) \left( \frac{\partial \theta }{\partial x} + \tau _{\theta } \frac{\partial ^2 \theta }{\partial t \partial x} \right) = 0, \end{aligned}$$
  5 $$\begin{aligned}{} & {} \tau _q \frac{\partial q_y}{\partial t} + q_y + K_{22}(\theta ) \left( \frac{\partial \theta }{\partial y} + \tau _{\theta } \frac{\partial ^2 \theta }{\partial t \partial y}\right) = 0, \end{aligned}$$
  where dependence of the heat conduction coefficients on temperature is taken into account (C.f^22^),
4. Generalized Hooke’s law differentiated w.r.t. time, thus allowing to replace the mechanical displacement components by those of velocity:
  6 $$\begin{aligned}{} & {} \frac{\partial \sigma _{xx}}{\partial t} - \left( \lambda + 2 \mu \right) \frac{\partial v_x}{\partial x} - \lambda \frac{\partial v_y}{\partial y} + \gamma \frac{\partial \theta }{\partial t} = 0, \end{aligned}$$
  7 $$\begin{aligned}{} & {} \frac{\partial \sigma _{yy}}{\partial t} - \lambda \frac{\partial v_x}{\partial x} - \left( \lambda + 2 \mu \right) \frac{\partial v_y}{\partial y} + \gamma \frac{\partial \theta }{\partial t} = 0, \end{aligned}$$
  8 $$\begin{aligned}{} & {} \frac{\partial \sigma _{xy}}{\partial t} - \mu \frac{\partial v_x}{\partial y} - \mu \frac{\partial v_y}{\partial x} = 0. \end{aligned}$$

These eight governing equations involve the velocity, stress, temperature and heat flux as basic unknowns. They are complemented, in case the displacements are needed, with the relations

9 $$\begin{aligned}{} & {} \frac{\partial u_x}{\partial t} - v_x = 0, \end{aligned}$$

10 $$\begin{aligned}{} & {} \frac{\partial u_y}{\partial t} - v_y = 0. \end{aligned}$$

As pointed above, nonlinearity is conditioned by the dependence of the heat conduction coefficients on temperature. Here, $\rho$ is the mass density, $\lambda , \mu -$ Lam$\acute{e}$ coefficients, $\gamma -$ the thermoelastic coefficient, $K_{11}, K_{22}-$ the coefficients of heat conduction and $\tau _q, \tau _{\theta }-$ the relaxation times related to temperature and heat flux respectively.

The following non-dimensional primed quantities are introduced for convenience in terms of some characteristic parameters:

$$\begin{aligned}{} & {} x=L_{0}{x}',\quad y=L_{0}{y}',\quad \theta =\Theta _{0}{\theta }',\quad t=\tau _{0}{t}',\quad u_{x}=L_{0}{u_{x}}',\quad u_{y}=L_{0}{u_{y}}',\quad q_{x}=Q_{0}{q_{x}}', \\{} & {} q_{y}=Q_{0}{q_{y}}',\quad v_{x}=\frac{L_{0}}{\tau _{0}}{v_{x}}',\quad v_{y}=\frac{L_{0}}{\tau _{0}}{v_{y}}',\quad \sigma _{xx}=\mu {\sigma _{xx}}',\quad \sigma _{xy}=\mu {\sigma _{xy}}',\quad \sigma _{yy}=\mu {\sigma _{yy}}',\\{} & {} K_{11}=K_{0}{K_{11} }',\quad K_{22}=K_{0}{K_{22} }',\quad {\tau _{q}}=\tau _{0}{\tau _{q} }',\quad {\tau _{\theta }}=\tau _{0}{\tau _{\theta } }', \end{aligned}$$

Moreover, define the dimensionless parameters $\beta _i, \, i=1,2, \cdots 4,$ in terms of the characteristic quantities by the relations:

$$\begin{aligned} \beta _1 = \frac{\mu \tau _{0} C_e}{K_0},\quad \beta _2 = \frac{\gamma }{ \rho C_{e} },\quad \beta _3 = \frac{\lambda }{\mu },\quad \beta _4 = \frac{\gamma \theta _{0} }{\mu },\quad \alpha =\frac{\lambda }{4 (\lambda +\mu )},\quad \beta =\frac{\lambda +2 \mu }{4 (\lambda +\mu )}. \end{aligned}$$

It is easy to show that

$$\begin{aligned} \alpha ^2 - \beta ^2 = \frac{1}{4 \mu \left( \lambda + \mu \right) } = \frac{\left( 1 + \nu \right) ^2 \left( 1 - 2 \nu \right) }{E} >0, \end{aligned}$$

where *E* and $\nu$ are Young’s modulus and Poisson’s ratio for the elastic material respectively, defined as:

11 $$\begin{aligned} E = \frac{\mu \left( 3 \lambda + 2 \mu \right) }{\lambda + \mu }, \qquad \nu = \frac{\lambda }{2 \left( \lambda +\mu \right) }. \end{aligned}$$

We shall take the characteristic quantities as:

$$\begin{aligned} \Theta _{0}=\theta _{0}, \quad L_{0}= \sqrt{\frac{\tau _0 K_0}{\rho C_e}},\quad Q_{0}=\Theta _{0} \sqrt{\frac{\rho C_e K_0 }{\tau _0 }}, \end{aligned}$$

by which it is seen that the characteristic velocity

$$ \frac{L_0}{T_0} = \sqrt{\frac{K_0}{\rho C_e}} \, \frac{1}{\sqrt{\tau _0}}$$

is closely related to the velocity of second sound.

Suppressing the primes, the dimensionless equations arising from Eqs.(1)-(8) are as follows:

12 $$\begin{aligned}{} & {} \frac{\partial v_x}{\partial t}-\beta _1\left( \frac{\partial \sigma _{xx}}{\partial x}+\frac{\partial \sigma _{xy}}{\partial y}\right) =0, \end{aligned}$$

13 $$\begin{aligned}{} & {} {\frac{\partial v_y}{\partial t}-\beta _1\left( \frac{\partial \sigma _{xy}}{\partial x}+\frac{\partial \sigma _{yy}}{\partial y}\right) =0,} \end{aligned}$$

14 $$\begin{aligned}{} & {} {\frac{\partial \theta }{\partial t}+\beta _2\left( \frac{\partial v_x}{\partial x}+\frac{\partial v_y}{\partial y}\right) + \frac{\partial \, q_x}{\partial x}+\frac{\partial \, q_y}{\partial y} =0,} \end{aligned}$$

15 $$\begin{aligned}{} & {} { \tau _q \frac{\partial q_x}{\partial t}+q_x+K_{11}(\theta )\left( \frac{\partial \theta }{\partial x}+\tau _{\theta } \frac{\partial ^2\theta }{\partial x\partial t}\right) =0,} \end{aligned}$$

16 $$\begin{aligned}{} & {} { \tau _q \frac{\partial q_y}{\partial t}+q_y+K_{22}(\theta )\left( \frac{\partial \theta }{\partial y}+ \tau _{\theta } \frac{\partial ^2\theta }{\partial y\partial t}\right) =0,} \end{aligned}$$

17 $$\begin{aligned}{} & {} {\frac{\partial \sigma _{xx}}{\partial t}- (\beta _3 +2) \frac{\partial v_x}{\partial x} - \beta _3 \frac{\partial v_y}{\partial y} +\beta _4 \frac{\partial \theta }{\partial t}=0,} \end{aligned}$$

18 $$\begin{aligned}{} & {} {\frac{\partial \sigma _{yy}}{\partial t}- \beta _3 \frac{\partial v_x}{\partial x} - (\beta _3 +2) \frac{\partial v_y}{\partial y} +\beta _4 \frac{\partial \theta }{\partial t}=0,} \end{aligned}$$

19 $$\begin{aligned}{} & {} {\frac{\partial \sigma _{xy}}{\partial t}-\frac{\partial v_x}{\partial y}-\frac{\partial v_y}{\partial x}=0.} \end{aligned}$$

Taking a transformation of the stress components:

20 $$\begin{aligned} \tilde{ \sigma }_{xx} = \sigma _{xx}+\beta _4 \, \theta , \qquad \tilde{\sigma }_{yy}= \sigma _{yy}+\beta _4 \, \theta , \qquad \tilde{\sigma }_{xy}= \sigma _{xy}, \end{aligned}$$

arranging the equations for the new stress components correspondingly and removing the “tilde”, one obtains:

21 $$\begin{aligned}{} & {} \frac{\partial v_x}{\partial t}-\beta _1\left( \frac{\partial \sigma _{xx}}{\partial x}-\beta _4\frac{\partial \theta }{\partial x}+\frac{\partial \sigma _{xy}}{\partial y}\right) =0, \end{aligned}$$

22 $$\begin{aligned}{} & {} {\frac{\partial v_y}{\partial t}-\beta _1\left( \frac{\partial \sigma _{xy}}{\partial x}-\beta _4\frac{\partial \theta }{\partial y}+\frac{\partial \sigma _{yy}}{\partial y}\right) =0,} \end{aligned}$$

23 $$\begin{aligned}{} & {} {\frac{\partial \theta }{\partial t}+\beta _2\left( \frac{\partial v_x}{\partial x}+\frac{\partial v_y}{\partial y}\right) +\left( \frac{\partial \, q_x}{\partial x}+\frac{\partial \, q_y}{\partial y}\right) =0,} \end{aligned}$$

24 $$\begin{aligned}{} & {} { \tau _q\frac{\partial q_x}{\partial t}+q_x+K_{11}(\theta ) \left( \frac{\partial \theta }{\partial x}+ \tau _{\theta } \frac{\partial ^2\theta }{\partial x\partial t}\right) =0,} \end{aligned}$$

25 $$\begin{aligned}{} & {} { \tau _q\frac{\partial q_y}{\partial t}+q_y+K_{22}(\theta )\left( \frac{\partial \theta }{\partial y}+ \tau _{\theta } \frac{\partial ^2\theta }{\partial y\partial t}\right) =0,} \end{aligned}$$

26 $$\begin{aligned}{} & {} {\frac{\partial }{\partial t}\left( \beta \sigma _{xx}-\alpha \sigma _{yy}\right) -\frac{\partial v_x}{\partial x}=0,} \end{aligned}$$

27 $$\begin{aligned}{} & {} {\frac{\partial }{\partial t}\left( -\alpha \sigma _{xx}+\beta \sigma _{yy}\right) -\frac{\partial v_y}{\partial y}=0,} \end{aligned}$$

28 $$\begin{aligned}{} & {} {\frac{\partial \sigma _{xy}}{\partial t}-\frac{\partial v_x}{\partial y}-\frac{\partial v_y}{\partial x}=0.}\ \end{aligned}$$

As we are considering Rayleigh wave propagation, this type of surface waves has an amplitude that is exponentially decreasing in depth into the medium. Following normal modes analysis, one may look for a particular solution for all the unknowns of the problem in the form:

29 $$\begin{aligned}{} & {} \left[ v_x, v_y, \theta , \sigma _{xx}, \sigma _{yy}, \sigma _{xy}, q_x, q_y \right] (x,y,t)  \nonumber \\{} & \quad = \varepsilon \left[ v^*_x, v^*_y, \theta ^*, \sigma ^*_{xx}, \sigma ^*_{yy}, \sigma ^*_{xy}, q^*_x, q^*_y \right] (y) \, e^{i(k x-\omega _i t) - \omega _r t} + \nonumber \\&\quad +   \varepsilon ^2 \left[ v^{**}_x, v^{**}_y, \theta ^{**}, \sigma ^{**}_{xx}, \sigma ^{**}_{yy}, \sigma ^{**}_{xy}, q^{**}_x, q^{**}_y \right] (y) \, e^{2i(k x - \omega _i t) -2 \omega _r t} + \cdots , \end{aligned}$$

where $$v^*_x, v^*_y, \theta ^*, \sigma ^*_{xx}, \sigma ^*_{yy}, \sigma ^*_{xy}, q^*_x, q^*_y, v^{**}_x, v^{**}_y, \theta ^{**}, \sigma ^{**}_{xx}, \sigma ^{**}_{yy}{}, \sigma ^{**}_{xy}, q^{**}_x $$ and $$q^{**}_y$$ are the amplitudes of the functions $$v_x, v_y, \theta , \sigma _{xx}, \sigma _{yy}, \sigma _{xy}, q_x $$ and $$q_y$$, respectively, and $$\varepsilon $$ is an adequately chosen small parameter representing the amplitude of variation of the temperature from the reference temperature $$\Theta _0$$. Here, *k* denotes the wavenumber, $$\omega _r$$-the frequency and $$\omega _i$$-the time damping, or attenuation coefficient. The dependence of some of the unknowns on the others will be taken into account in what follows. We shall be interested in the first two orders of approximation, as it has been revealed in the literature that second harmonics play a fundamental role in the response of the medium to applied loads. In^7,17^, the authors use the dual-phase-lag model to study the propagation of linear Rayleigh waves in a half-space under various mechanical and thermal boundary conditions. A frequency equation is derived and the effect of thermal relaxation times is considered. In the present work, the boundary conditions are different: prescribed normal mechanical load and temperature, and we proceed otherwise. In order to concentrate our attention on the nonlinearity of the equations, we simply find particular solutions for the linear approximation for selected wave number, frequency and attenuationg coefficient. The influence on surface wave propagation of the linear dependence of the heat conduction coefficient on temperature, as well as thermal relaxation times, is investigated.

Substituting from Eq. (29) into Eqs. (21)–(28), and denoting $D= \frac{d}{dy}$, we get a system of homogeneous linear ordinary differential equations of the first order, and a system of non-homogeneous linear ordinary differential equations of the first order, in the first two orders of approximation respectively. In this procedure, we have set

30 $$\begin{aligned} K_{11} \left( \theta \right) =K_{22} \left( \theta \right) =K_{0}(1+\eta \, \theta ), \end{aligned}$$

thus allowing for a linear dependence of the heat conduction coefficient on temperature. As noted above, such a dependence may be relevant, especially at high temperatures and in nano-structures, when the material characteristics can no longer be considered as constants (C.f.^22,30^). The introduced parameter $\eta$ has order of magnitude equal to unity. The linear dependence of the thermal conductivity on temperature is effective when the changes of temperature are small enough, an has been considered in many cases (C.f.^24,26–30^). Other forms of dependence on temperature may be found in^23,25^.

The system of homogeneous linear differential equations of the first order:

31 $$\begin{aligned} D v ^*_x&=   A_1 \, v ^*_y +A_2 \, \sigma _{xy}^*, \end{aligned}$$

32 $$\begin{aligned} D v ^*_y&=   A_{3} \, v ^*_x +A_4 \, \sigma ^*_{yy}, \end{aligned}$$

33 $$\begin{aligned} D \theta ^*&=   A_5 \, q^*_y, \end{aligned}$$

34 $$\begin{aligned} D \sigma ^*_{yy}&=   A_{6} \, v ^*_y +A_1 \, \sigma ^*_{xy} +A_7 \, q^*_y, \end{aligned}$$

35 $$\begin{aligned} D \sigma ^*_{xy}&=   A_8 \, v ^*_x + A_9 \, \theta ^*+A_3\sigma ^*_{yy}, \end{aligned}$$

36 $$\begin{aligned} D q^*_y&=   A_{10} \, \theta ^*+A_{11} \, v ^*_x +A_{12} \, \sigma ^*_{yy} , \end{aligned}$$

together with

37 $$\begin{aligned} \sigma ^*_{xx}&=   A_{13} \, v ^*_x +A_{14 } \, \sigma ^*_{yy}, \end{aligned}$$

38 $$\begin{aligned} q^*_x&=   A_{15 } \, \theta ^* . \end{aligned}$$

The system of non-homogeneous linear differential equations of the first order:

39 $$\begin{aligned} D v ^{**}_x&=   A_{16} \, v ^{**}_y +A_{17} \, \sigma ^{**}_{xy}, \end{aligned}$$

40 $$\begin{aligned} D v ^{**}_y&=   A_{18} \, v ^{**}_x + A_{19} \, \sigma ^{**}_{yy}, \end{aligned}$$

41 $$\begin{aligned} D \theta ^{**}&=   A_{20} \, q^{**}_y +A_{31} \, \theta ^* D \theta ^*, \end{aligned}$$

42 $$\begin{aligned} D \sigma ^{**}_{yy}&=   A_{21} \, v ^{**}_y +A_{16} \, \sigma ^{**}_{xy} +A_{22}q^{**}_y +A_{32} \theta ^* D \theta ^*, \end{aligned}$$

43 $$\begin{aligned} D \sigma ^{**}_{xy}&=   A_{23} \, v ^{**}_x +A_{24} \, \theta ^{**}+A_{18} \, \sigma ^{**}_{yy}, \end{aligned}$$

44 $$\begin{aligned} D q^{**}_y&=   A_{25 } \, \theta ^{**}+A_{26} \, v ^{**}_x +A_{27} \, \sigma ^{**}_{yy} + A_{33} \, \theta ^{*2} , \end{aligned}$$

together with

45 $$\begin{aligned} \sigma ^{**}_{xx}&=   A_{28} v ^{**}_x +A_{29}\sigma ^{**}_{yy}, \end{aligned}$$

46 $$\begin{aligned} q^{**}_x&=   A_{30}\theta ^{**}+A_{34} \theta ^{*2}. \end{aligned}$$

and $$A_{j},j=1,2,...,34 $$ are constants listed in "Appendix A". It clearly appears that quadratic expressions in $$\theta ^*$$ will be responsible for the generation of the solution at the second order of approximation.

## Solution of the problem

### The homogeneous system

47 $$\begin{aligned} {\left( \begin{array}{c} D v_x^* \\ D v_y^* \\ D \theta ^* \\ D \sigma _{yy}^* \\ D \sigma _{xy}^* \\ D q_y^* \\ \end{array} \right) =\left( \begin{array}{cccccc} 0 &{} A_1 &{} 0 &{} 0 &{} A_2 &{} 0 \\ A_3 &{} 0 &{} 0 &{} A_4 &{} 0 &{} 0 \\ 0 &{} 0 &{} 0 &{} 0 &{} 0 &{} A_5 \\ 0 &{} A_6 &{} 0 &{} 0 &{} A_1 &{} A_7 \\ A_8 &{} 0 &{} A_9 &{} A_3 &{} 0 &{} 0 \\ A_{10} &{} 0 &{} A_{11} &{} A_{12} &{} 0 &{} 0\text { } \\ \end{array} \right) \left( \begin{array}{c} v_x^* \\ v_y^* \\ \theta ^* \\ \sigma _{yy}^* \\ \sigma _{xy}^* \\ q_y^* \\ \end{array} \right) .} \end{aligned}$$

Assuming a solution of the form $e^{\xi y}$, the characteristic equation for the eigenvalues $\xi$ for this system of equations is obtained as:

48 $$\begin{aligned} \xi ^{6} - A \xi ^{4} + B \xi ^{2} - C = 0, \end{aligned}$$

where A, B and C are constants listed in "Appendix A".

Only three roots of this equation, $\xi _n, n=1,2,3$ with positive real parts, will contribute to the bounded solution. Following Eq. (48), any imaginary part of $\xi$ will result in a circular function of sine or cosine in the solution, i.e. an amplitude that is oscillating in y while damped exponentially in y. In the end, only the real part of the solution will have physical meaning.The solution of the system of equations (47) may be written conveniently in the form:

49 $$\begin{aligned}{} & {} {v_x^*=\sum _{n=1}^3 \text { }v_{1n} \,M_{n } e^{-\xi _ny},} \end{aligned}$$

50 $$\begin{aligned}{} & {} {v_y^*=\sum _{n=1}^3 v_{2 n} \, M_{n } e^{-\xi _ny},} \end{aligned}$$

51 $$\begin{aligned}{} & {} {\theta ^*=\sum _{n=1}^3 v_{3 n} \, M_{n } e^{-\xi _ny},} \end{aligned}$$

52 $$\begin{aligned}{} & {} {\sigma _{yy}^*=\sum _{n=1}^3 v_{4n} \, M_{n } e^{-\xi _ny},} \end{aligned}$$

53 $$\begin{aligned}{} & {} {\sigma _{xy}^*=\sum _{n=1}^3 v_{5 n} \, M_{n } e^{-\xi _ny},} \end{aligned}$$

54 $$\begin{aligned}{} & {} {q_y^*=\sum _{n=1}^3 v_{6n} \, M_{n }e^{-\xi _ny}.} \end{aligned}$$

where $v_{mn}, m=1,2,...,6, \, n=1,2,3$ are constants listed in "Appendix B". The remaining two solution functions may now be calculated from Eqs.(37) and (38).

#### Boundary conditions for the homogeneous system

Generally, the thermal boundary conditions fall into three categories: Either temperature is prescribed, or heat flux, or else a radiation-type condition involving both. As to the mechanical boundary conditions, they concern either the displacements (e.g. fixed boundary), or the stresses (stress-free or prescribed load). For definiteness, it is assumed that temperature is prescribed at the boundary of the material, and that this boundary is subjected to a normal mechanical load^14^:

1. Mechanical boundary conditions:
  55 $$\begin{aligned} \sigma _{yy}(x,0,t)=f_{1}(x,t)=-f_{1}^{*}e^{i\left( k x-\omega _r t\right) +\omega _i t},\qquad \sigma _{xy}(x,0,t)=0, \end{aligned}$$
2. Thermal boundary condition:
  56 $$\begin{aligned} \theta (x,0,t)=f_{2}(x,t)=f_{2}^{*}e^{i\left( k x-\omega _r t\right) +\omega _i t}. \end{aligned}$$

Other types of boundary conditions may be found elsewhere^17^.

Applying the boundary conditions (55) and (56), one determines the coefficients $M_{n}, \, n=1,2,3$ from the system of linear algebraic equations:

57 $$\begin{aligned}{} & {} \underset{n=1}{\overset{3}{\sum }}v_{4n} \, M_{n}=-f_{1}^{*}, \end{aligned}$$

58 $$\begin{aligned}{} & {} \underset{n=1}{\overset{3}{\sum }}v_{5n} \, M_{n}=0, \end{aligned}$$

59 $$\begin{aligned}{} & {} \underset{n=1}{\overset{2}{\sum }}v_{3n} \, M_{n}=f_{2}^{*}, \end{aligned}$$

Solving equations (57)-(59) for $$ M_{n}, n=1,2,3$$ by the inverse matrix method, one gets:

60 $$\begin{aligned} \left( \begin{array}{c} M_{1} \\ M_{2} \\ M_{3} \end{array} \right) =\left( \begin{array}{cccc} v_{41} &{} v_{42} &{} v_{43} \\ v_{51} &{} v_{52} &{} v_{53} \\ v_{31} &{} v_{32} &{} v_{33} \end{array} \right) ^{-1}\left( \begin{array}{c} -f_{1}^{*} \\ 0 \\ f_{2}^{*} \\ \end{array} \right) \end{aligned}$$

### The non-homogeneous system

$$\begin{aligned} {\left( \begin{array}{c} D v_x^{\text {**}} \\ D v_y^{\text {**}} \\ D \theta ^{\text {**}} \\ D \sigma _{yy}^{\text {**}} \\ D \sigma _{xy}^{\text {**}} \\ D q_y^{\text {**}} \\ \end{array} \right) =\left( \begin{array}{cccccc} 0 &{} A_{16} &{} 0 &{} 0 &{} A_{17} &{} 0 \\ A_{18} &{} 0 &{} 0 &{} A_{19} &{} 0 &{} 0 \\ 0 &{} 0 &{} 0 &{} 0 &{} 0 &{} A_{20} \\ 0 &{} A_{21} &{} 0 &{} 0 &{} A_{16} &{} A_{22} \\ A_{23} &{} 0 &{} A_{24} &{} A_{18} &{} 0 &{} 0 \\ A_{25} &{} 0 &{} A_{26} &{} A_{27} &{} 0 &{} 0\text { } \\ \end{array} \right) \left( \begin{array}{c} v_x^{\text {**}} \\ v_y^{\text {**}} \\ \theta ^{\text {**}} \\ \sigma _{yy}^{\text {**}} \\ \sigma _{xy}^{\text {**}} \\ q_y^{\text {**}} \\ \end{array} \right) +\left( \begin{array}{c} 0 \\ 0 \\ A_{31} \theta ^{*} {\theta ^{*}}' \\ A_{32} \theta ^{*} {\theta ^{*}}' \\ 0 \\ A_{33} {\theta ^{*}}{}^2 \\ \end{array} \right) } \end{aligned}$$

or

61 $$\begin{aligned} \left( \begin{array}{c} D v_x^{\text {**}} \\ D v_y^{\text {**}} \\ D \theta ^{\text {**}} \\ D \sigma _{yy}^{\text {**}} \\ D \sigma _{xy}^{\text {**}} \\ D q_y^{\text {**}} \\ \end{array} \right)= & {} \left( \begin{array}{cccccc} 0 &{} A_{16} &{} 0 &{} 0 &{} A_{17} &{} 0 \\ A_{18} &{} 0 &{} 0 &{} A_{19} &{} 0 &{} 0 \\ 0 &{} 0 &{} 0 &{} 0 &{} 0 &{} A_{20} \\ 0 &{} A_{21} &{} 0 &{} 0 &{} A_{16} &{} A_{22} \\ A_{23} &{} 0 &{} A_{24} &{} A_{18} &{} 0 &{} 0 \\ A_{25} &{} 0 &{} A_{26} &{} A_{27} &{} 0 &{} 0\text { } \\ \end{array} \right) \left( \begin{array}{c} v_x^{\text {**}} \\ v_y^{\text {**}} \\ \theta ^{\text {**}} \\ \sigma _{yy}^{\text {**}} \\ \sigma _{xy}^{\text {**}} \\ q_y^{\text {**}} \\ \end{array} \right) \nonumber \\{} & {} - \left( \begin{array}{c} 0 \\ 0 \\ \sum _{i=1}^3 \sum _{j=1}^3 A_{31} \xi _i v_{3 i} v_{3 j} M_i M_j \, e^{-y \left( \xi _i+\xi _j\right) } \\ \sum _{i=1}^3 \sum _{j=1}^3 A_{32} \xi _i v_{3 i} v_{3 j} M_i M_j \, e^{-y \left( \xi _i+\xi _j\right) } \\ 0 \\ \sum _{i=1}^3 \sum _{j=1}^3 -A_{33} v_{3 i} v_{3 j}M_i M_j \, e^{-y \left( \xi _i+\xi _j\right) } \\ \end{array} \right) \end{aligned}$$

Solving the homogenuous part of Eq. (61), one writes down the characteristic polynomial as:

62 $$\begin{aligned} \zeta ^6- B_1 \zeta ^4+B_2 \zeta ^2-B_3=0 , \end{aligned}$$

where $B_1, B_2$ and $B_3$ are constants listed in "Appendix A".

As for the first order solution, only three roots, $\zeta _n, n=1,2,3$ will contribute to the bounded solution of Eq. (61). Now solve for the non-homogeneous part by the method of undetermined coefficients. The particular solution is taken in the form:

63 $$\begin{aligned} \sum _{i=1}^3 \sum _{j=1}^3 E_{n,ij} v_{3 i} v_{3 j} \, M_i M_j \, e^{-y \left( \xi _i+\xi _j\right) }, \quad n=1,2, \cdots , 6. \end{aligned}$$

Substitution of this expression into the system yields the coefficients $E_{n,ij}$:

$$\begin{aligned} { \begin{pmatrix} E_{1,ij} \\ E_{2,ij}\\ E_{3,ij} \\ E_{4,ij} \\ E_{5,ij} \\ E_{6,ij} \end{pmatrix} \text {=}\left( \begin{array}{cccccc} \xi _i+\xi _j &{} A_{16} &{} 0 &{} 0 &{} A_{17} &{} 0 \\ A_{18} &{} \xi _i+\xi _j &{} 0 &{} A_{19} &{} 0 &{} 0 \\ 0 &{} 0 &{} \xi _i+\xi _j &{} 0 &{} 0 &{} A_{20} \\ 0 &{} A_{21} &{} 0 &{} \xi _i+\xi _j &{} A_{16} &{} A_{22} \\ A_{23} &{} 0 &{} A_{24} &{} A_{18} &{} \xi _i+\xi _j &{} 0 \\ A_{25} &{} 0 &{} A_{26} &{} A_{27} &{} 0 &{} \xi _i+\xi _j\text { } \\ \end{array} \right) ^{-1}\left( \begin{array}{c} 0 \\ 0 \\ A_{31} \xi _i\\ A_{32} \xi _i\\ 0 \\ -A_{33} \\ \end{array} \right) }. \end{aligned}$$

Hence the solution of the system of equations (61) has the form:

64 $$\begin{aligned}{} & {} {v_x^{\text {**}}=\sum _{i=1}^3 \text { }V_{1 i}\text { }L_i\text { }e^{\zeta _iy}+\sum _{i=1}^3 \sum _{j=1}^3 E_{1,ij} v_{3 i} v_{3 j} M_i M_j \, e^{-\left( \xi _i+\xi _j\right) y}} \end{aligned}$$

65 $$\begin{aligned}{} & {} {v_y^{\text {**}}=\sum _{i=1}^3 V_{2i} L_i\text { }e^{\zeta _iy}+\sum _{i=1}^3 \sum _{j=1}^3 E_{2,ij}\text { }v_{3 i} v_{3 j} M_i M_j \, e^{-\left( \xi _i+\xi _j\right) y}} \end{aligned}$$

66 $$\begin{aligned}{} & {} {\theta ^{\text {**}}=\sum _{i=1}^3 V_{3 i} L_i\text { }e^{\zeta _iy}+\sum _{i=1}^3 \sum _{j=1}^3 E_{3,ij}\text { }v_{3 i} v_{3 j} M_i M_j \, e^{-\left( \xi _i+\xi _j\right) y}} \end{aligned}$$

67 $$\begin{aligned}{} & {} {\sigma _{yy}^{\text {**}}=\sum _{i=1}^3 V_{4 i} L_i\text { }e^{\zeta _iy}+\sum _{i=1}^3 \sum _{j=1}^3 E_{4,ij}\text { }v_{3 i} v_{3 j} M_i M_j \, e^{-\left( \xi _i+\xi _j\right) y}} \end{aligned}$$

68 $$\begin{aligned}{} & {} {\sigma _{xy}^{\text {**}}=\sum _{i=1}^3 V_{5 i} L_i\text { }e^{\zeta _iy}+\sum _{i=1}^3 \sum _{j=1}^3 E_{5,ij}\text { } v_{3 i} v_{3 j} M_i M_j \, e^{-\left( \xi _i+\xi _j\right) y}} \end{aligned}$$

69 $$\begin{aligned}{} & {} {q_y^{\text {**}}=\sum _{i=1}^3 V_{6 i} L_i\text { }e^{\zeta _iy}+\sum _{i=1}^3 \sum _{j=1}^3 E_{6,ij}\text { }v_{3 i} v_{3 j} M_i M_j \, e^{-\left( \xi _i+\xi _j\right) y}} \end{aligned}$$

where $V_{nj}, \, n=1,2,...,6, \, j=1,2,3$ are constants listed in "Appendix C".

#### Boundary conditions for the non-homogeneous system

Here we have taken vanishing boundary conditions, the solution at this order of approximation being generated solely by the non-homogeneous term originating from the previous order of approximation.

1. Mechanical boundary conditions:
  70 $$\begin{aligned} \sigma _{yy}(x,0,t)=0,\qquad \sigma _{xy}(x,0,t)=0, \end{aligned}$$
2. Thermal boundary condition:
  71 $$\begin{aligned} \theta (x,0,t)=0. \end{aligned}$$

Applying the boundary conditions (70)-(71) one determines the coefficients $L_{i}, \, i=1,2,3$ from the system of linear algebraic equations:

72 $$\begin{aligned}{} & {} { \sum _{i=1}^3 L_{i } V_{4n}+ \sum _{i=1}^3 \underset{j=1}{\overset{3}{\sum }} E_{4,ij} \, v_{3 i} v_{3 j} M_i M_j=0}, \end{aligned}$$

73 $$\begin{aligned}{} & {} {\sum _{i=1}^3 \text { }L_i V_{3n}+ \sum _{i=1}^3 \sum _{j=1}^3 \text { }E_{3,ij} \, v_{3 i} v_{3 j} M_i M_j =0}, \end{aligned}$$

74 $$\begin{aligned}{} & {} {\sum _{i=1}^3 L_i V_{5n}+\sum _{i=1}^3 \sum _{j=1}^3 E_{5,ij} \, v_{3 i} v_{3 j} M_i M_j=0 }. \end{aligned}$$

## Numerical results and discussion

This section is devoted to the analysis of a concrete numerical example. The following values were taken for the prescribed temperature and the pressure at the boundary:

$$ f^*_1=6.0, \qquad f^*_2=2.0.$$

These two values will be kept constant, while the coefficient of linear dependence of the heat conduction coefficient on temperature, named $\eta$, as well as the thermal relaxation times, will be varied. For the treated example, we have chosen the Cadmium Selenide (CdSe) material with hexagonal symmetry (6 mm class). The coordinate *z*-axis was oriented perpendicular to the plane of symmetry. The corresponding values of the material parameters are shown in Table 1 (C.f.^14^):

**Table 1:** Values of the geometrical and the material parameters.

$\Theta _0=298 \, K$	$K_0=9 \, W m^{-1}.K^{-1}$	
$\rho =5504 \, \text {kg}\,\text {m}^{-3}$,	$C_e =260 \, \text {J}\,\text {kg}^{-1}\,\text {K}^{-1}$	$\gamma =0.5 \times 10^5 \, \text {m}^3\,\text {kg}^{-1}$
$\lambda =2.6 \times 10^{10} \, \text {kg}\,\text {m}^{-1}\,\text {s}^{-2}$	$\mu =1.6 \times 10^{10} \, \text {kg}\,\text {m}^{-1}\,\text {s}^{-2}$	
$\tau _0=1.000 \times 10^{-11}$s	$\tau _{\theta }=0.500 \times 10^{-11}$ s	$\tau _q=0.600 \times 10^{-11}$ s
$k=0.2$ (wave number)	$\omega _r=0.5$	$\omega _i=0.5$

It is worth noting that the values of the thermal relaxation times appearing in this Table are only tentative, as these material constants do not seem to have well established values in the available literature.

**Figure 2:**
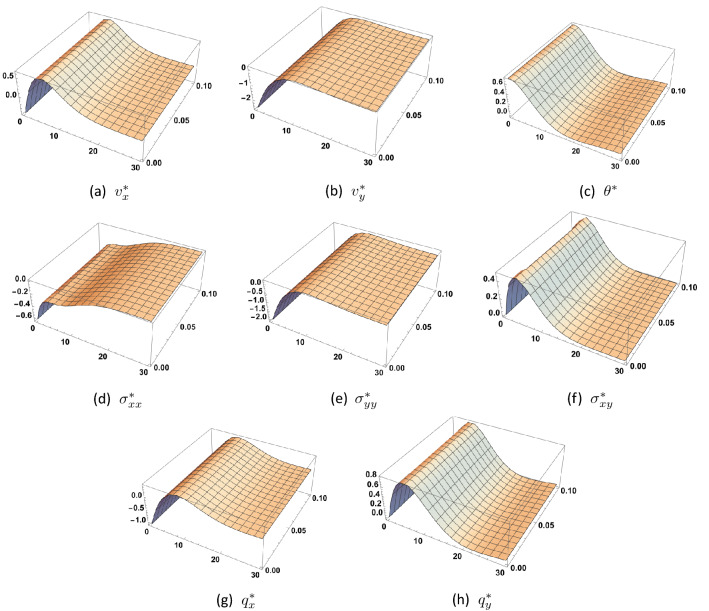
First order solution as function of (*x*, *y*) at $t=1.5$ and for $\tau _{q}=0.6$, $\tau _{\theta }=0.5$.

Figure 2 illustrates the 3*D*-solution at the first order of approximation (the linear problem) as function of the coordinates (*x*, *y*) for $0 \le y \le 30, \, 0 \le x \le 0.1$ and $t=1.5$. As expected, the amplitudes of all the solution functions asymptotically decrease exponentially with depth. Obviously, the same holds true for the solution in the next order of approximation. Thus, any experimental detection of these harmonics (basic or higher) is bound to happen near the surface of the specimen.

**Figure 3:**
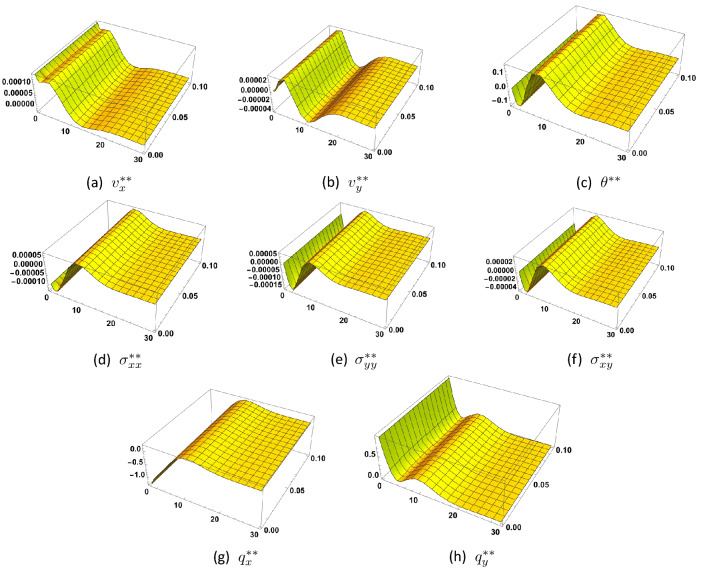
Second order solution as function of (*x*, *y*) at $t=1.5$, and for $\tau _{q}=0.6$, $\tau _{\theta }=0.5$, $\eta =1.0$.

Figure 3 shows the 3*D*-solution at the second order of approximation as function of the coordinates (*x*, *y*) for $0 \le y \le 30, \, 0 \le x \le 0.1$ and $t=1.5$. Comparing these figures with those of the first order approximation, we note that the behavior of temperature and normal stress has changed because of the different boundary conditions imposed on them, while the heat flux components retain the same type of behavior.

Referring to Figs. 2 and 3, one sees that the solution nearly vanishes for $y > 10$. We may thus deduce that there is a slab at the surface of the material beyond which all amplitudes are practically equal to zero. This slab has dimensionless thickness $L = 10$. For the used numerical values of the material coefficients, frequency and wavenumber, the thickness of this slab, also called penetration depth, is of the order of $2.379 \times 10^{-7} m$. Variation of the penetration depth with wavenumber may be found in^13^.

**Figure 4:**
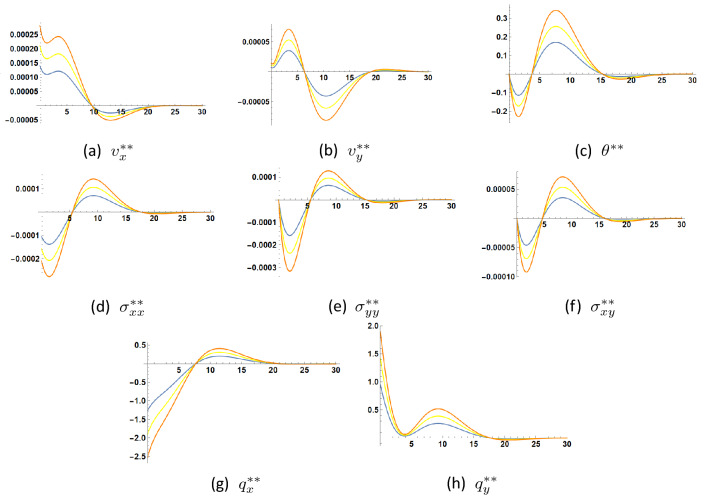
Second order solution as function of *y* at $x=0.1$, $t=1.5$ for three values of $\eta.$ $\eta =1$ $\eta =1.5$ $\eta =2$.

**Figure 5:**
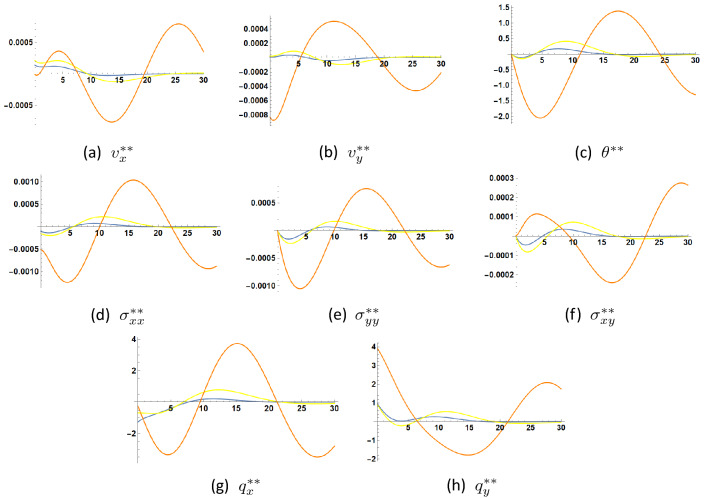
Second order solution as function of *y* at $x=0.1$, $t=1.5$ for $\tau _{\theta }=0.5$ and three values of $\tau _q$ $\tau _q=0.6$ $\tau _q=0.8$ $\tau _q=1.0$.

**Figure 6:**
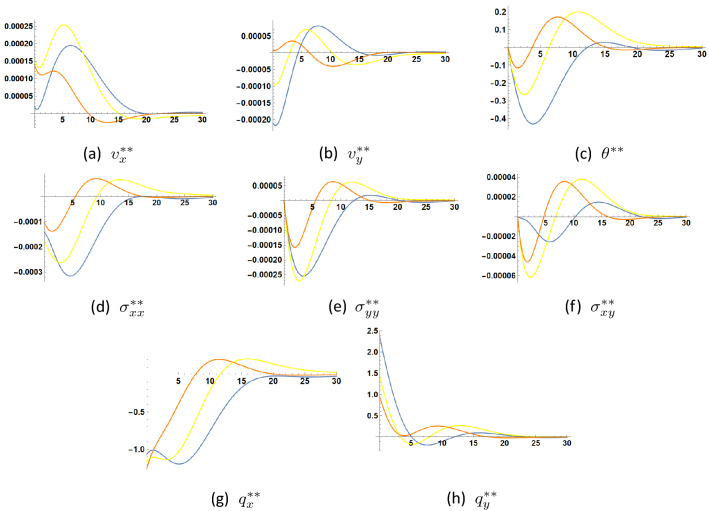
Second order solution as function of *y* at $x=0.1$, $t=1.5$ for $\tau _q =0.6$ and three values of $\tau _{\theta }$ $\tau _{\theta }=0.1$ $\tau _{\theta }=0.3$ $\tau _{\theta }=0.5$.

Figures 4, 5 and 6 illustrate the changes occuring in the amplitudes of the second order harmonic when the coefficients $\eta , \tau _{\theta }$ and $\tau _q$ are varied. Such changes can be appreciable and may be experimentally detected through experimental measurements of the mechanical displacements or velocities (C.f.^1^) to assess the nonlinear contribution of parameter $\eta$ expressing the linear dependence of the heat conduction coefficient on temperature and the two thermal relaxation parameters $\tau _{\theta }$ and $\tau _q$.

In Fig. 4 it is seen that the increase of parameter $\eta$ increases the fluctuations of the unknown functions. These fluctuations indicate the presence of imaginary parts in the roots $\zeta _n$. The proposed formulae for the solution allow to carry out the detection of the second harmonic amplitudes through the measurement of several quantities: temperature, mechanical displacements or velocities, heat flux and stress.

The same can be said about the increase of parameter $\tau _q$ as shown in Fig. 5.

As to Fig. 6, it is inconclusive in what concerns the effect of increase of parameter $\tau _{\theta }$. It decreases the fluctuations in both stress components $\sigma _{xx}$ and $\sigma _{yy}$ but decreases them in $\sigma _{xy}$. Damping in temperature is observed as $\tau _{\theta }$ increases.

Comparison shows that the orders of magnitude of temperature and heat flux in the first two approximations differ at most by one order of magnitude, while the velocities and stresses in the second approximation are three to four orders of magnitude less than their counterparts in the first approximation. It thus becomes clear that it is advantageous to use thermal measurements to detect the higher harmonic solution, rather than velocity or stress measurements.

The present work may be considered as an extension of^7^ to the nonlinear case. Although the governing equations are the same as those used here, the materials and the initial and boundary conditions are different: The present work has no initial conditions, and the boundary is subjected to given temperature and normal stress loads, while the motion investigated in^7^ is generated by an incoming rotational wave, with zero mechanical load and a vanishing normal derivative of temperature at the boundary. This makes it difficult to establish a comparison between the numerical results, but qualitative results that “the effect of the phase-lag of the heat flux $\tau _q$ and the phase-lag of gradient of temperature $\tau _{\theta }$ is significantly clear on Rayleigh waves and the amplitudes of the displacements, temperature and thermal stresses”  are in agreement.

As to the work in^27^, it presents a finite element formulation for nonlinear surface wave propagation in a half-space of an isotropic thermoelastic diffusive solid in extended thermodynamics with only one thermal relaxation time. This may be derived from our model by setting $$\tau _{\theta }=0$$. Motion in this reference starts from rest under a variable temperature and zero mechanical load at the boundary. The same linear dependence of thermal conductivity on temperature is considered as for the present work. Again, it is difficult to compare the numerical results in^27^ with those presented here in view of the difference of material coefficients, as well as the used initial and boundary conditions. In this reference, it is concluded that “the variable thermal conductivity has an increasing effect on the distributions of temperature, displacement and stress”. In the present case, such a conclusion could be verified only in certain cases as suggested by Figs. 3 and 4, in which one notices a positive second order solution for the function $$v_x^{**}$$, while the other functions may assume positive or negative values according to depth location. Other different configurations are possible for different time moments. However, as noted above, the fluctuations of the distributions of all unknown functions tend to increase with increasing coefficient of linearity $$\eta $$.

## Conclusions

We have investigated the propagation of nonlinear Rayleigh waves in a half-space of a transversely isotropic thermoelastic material within dual-phase-lag theory. The model allows for heat wave propagation.The aim of the work is to propose a method for evaluating the coefficient of linearity in the dependence of the thermal conductivity on temperature, through the measurement of some quantities of practical interest at the surface of the specimen, caused by Rayleigh wave propagation. The boundary of the medium was subjected to a prescribed temperature and a normal mechanical load. Nonlinearity arises from a linear dependence of the heat conduction coefficients on temperature. Such a dependence of the material coefficients on temperature is known to be relevant for an efficient description of the continuum at higher temperatures and in nano-structures. The governing equations are presented in the form of a system of first order partial differential equations, which is advantageous for numerical study. A particular solution is obtained using the normal modes technique in the first two orders of approximation only. The results are discussed in detail and the quantities of practical interest are represented graphically in two-and three-dimensional plots. In particular, we have extended existing work of the linear theory to assess the influence of the temperature dependence of the heat conduction coefficients, and the thermal relaxation times on nonlinear wave propagation. The numerical results show that these parameters can produce appreciable changes in the amplitudes of the generated higher harmonics, and thus can be detected experimentally through the measurement of temperature, velocities, heat flux or stresses. However, it is advantageous to use thermal measurements as the higher order amplitudes of temperature and heat flux have appreciable values as shown by the figures. In addition, the measured nonlinearity is closely related to cumulative plastic deformation, showing that the obtained results may be of interest in characterizing the damage associated with plastic deformation.

All calculations were performed on Mathematica 12.3 Software.

## Supplementary Information

Supplementary Information 1.

## Data Availability

All data generated or analyzed during this study are included in this published article.
