# Supplementary material for: Nonlinear Rayleigh wave propagation in thermoelastic media in dual-phase-lag
Source: Sci Rep. 2022 Dec 8;12:21209. doi: 10.1038/s41598-022-25680-7 (PMC9731999; doi:10.1038/s41598-022-25680-7)
Supplement: Supplementary file 1 — Supplementary Information 1. [file 41598_2022_25680_MOESM1_ESM.pdf]

Appendix A

Coefficients 1

$A_1 = -ik, A_2 = -\omega_r - i\omega_i, A_3 = \frac{-ik\alpha}{\beta}, A_4 = \frac{(\alpha-\beta)(\alpha+\beta)(\omega_r+i\omega_i)}{\beta},$

$A_5 = \frac{1-(\omega_r+i\omega_i)\tau_q}{K_0(-1+(\omega_r+i\omega_i)\tau_\theta)}, A_6 = -\frac{(\omega_r+i\omega_i)}{\beta_1}, A_7 = -\frac{\beta_4(-1+(\omega_r+i\omega_i)\tau_q)}{K_0(-1+(\omega_r+i\omega_i)\tau_\theta)},$

$A_8 = -\frac{k^2}{\beta(\omega_r+i\omega_i)} - \frac{(\omega_r+i\omega_i)}{\beta_1}, A_9 = ik\beta_4, A_{11} = \left((\omega_r + i\omega_i) - \frac{k^2K_0(-1+(\omega_r+i\omega_i)\tau_\theta)}{-1+(\omega_r+i\omega_i)\tau_q}\right),$

$A_{10} = \frac{(\alpha-\beta)\beta_2(ik)}{\beta}, A_{12} = \frac{(-\alpha+\beta)(\alpha+\beta)(\omega_r+i\omega_i)\beta_2}{\beta}, A_{13} = \frac{k}{\beta(i\omega_r-\omega_i)}, A_{14} = \frac{\alpha}{\beta},$

$A_{15} = \frac{kK_0(i+(-i\omega_r+\omega_i)\tau_\theta)}{-1+(\omega_r+i\omega_i)\tau_q}, A_{16} = -i2k, A_{17} = -2\left(\omega_r + i\omega_i\right), A_{18} = -\frac{i2\ k\alpha}{\beta},$

$A_{19} = \left(-2\beta\left(\omega_r + i\omega_i\right)\right) + \frac{\alpha(2\alpha(\omega_r+i\omega_i))}{\beta}, A_{20} = \frac{(1-2(\omega_r+i\omega_i)\tau_q)}{K_0(-1+2(\omega_r+i\omega_i)\tau_\theta)},$

$A_{21} = -\frac{2(\omega_r+i\omega_i)}{\beta_1}, A_{22} = \frac{\beta_4(1-2(\omega_r+i\omega_i)\tau_q)}{K_0(-1+2(\omega_r+i\omega_i)\tau_\theta)}, A_{23} = -\frac{2k^2}{\beta(\omega_r+i\omega_i)} - \frac{2(\omega_r+i\omega_i)}{\beta_1},$

$A_{24} = 2ik\beta_4, A_{25} = \frac{2ik(\alpha-\beta)\beta_2}{\beta}, A_{26} = 2\left(\omega_r + i\omega_i\right) - \frac{4k^2K_0(-1+2(\omega_r+i\omega_i)\tau_\theta)}{-1+2(\omega_r+i\omega_i)\tau_q},$

$A_{27} = -\frac{2\beta_2(\alpha-\beta)(\alpha+\beta)(\omega_r+i\omega_i)}{\beta}, A_{28} = -\frac{ik}{\beta(\omega_r+i\omega_i)}, A_{29} = \frac{\alpha}{\beta},$

$A_{30} = \frac{2kK_0(1+\tau_\theta(-2i\omega_i-2\omega_r))}{i+2\tau_q(\omega_i-i\omega_r)}, A_{31} = \eta\frac{1}{2}\left(-1 + \frac{1}{-1+2\tau_\theta(i\omega_i+\omega_r)}\right), A_{32} = \eta\frac{1}{2}\beta_4\left(-1 + \frac{1}{-1+2\tau_\theta(i\omega_i+\omega_r)}\right),$

$A_{33} = -\eta\frac{2k^2K_0(-1+\tau_\theta(i\omega_i+\omega_r))}{-1+2\tau_q(i\omega_i+\omega_r)}, A_{34} = \eta\frac{kK_0(1+\tau_\theta(-i\omega_i-\omega_r))}{i+2\tau_q(\omega_i-i\omega_r)},$

$A = \left(2A_1A_3 + A_{11}A_5 + A_4A_6 + A_{12}A_7 + A_2A_8\right),$

$B = A_1^2\left(A_3^2 - A_4A_8\right) + A_4A_5A_6A_{11} + A_1\left(2A_3A_5A_{11} - A_4A_7A_{10} + \left(A_3A_7 - A_5A_9\right)A_{12}\right)$

$-A_2\left(A_3^2A_6 + A_3A_7A_{10} + A_5A_9A_{10} - A_8\left(A_4A_6 + A_5A_{11} + A_7A_{12}\right)\right),$

$C = A_5\left({A_1}^2 - A_2A_6\right)\left(A_{11}\left({A_3}^2 - A_4A_8\right) - A_{12}A_3A_9 + A_{10}A_4A_9\right),$

$B_1 = \left(2A_{16}A_{18} + A_{26}A_{20} + A_{19}A_{21} + A_{27}A_{22} + A_{17}A_{24}\right),$

$B_2 = A_{26}A_{19}A_{20}A_{21} + {A_{16}}^2\left({A_{18}}^2 - A_{19}A_{24}\right)$

$-A_{17}\left({A_{18}}^2A_{21} + A_{25}A_{18}A_{22} - \left(A_{26}A_{20} + A_{19}A_{21} + A_{27}A_{22}\right)A_{24} + A_{25}A_{20}\ A_{24}\right)$

$+A_{16}\left(2A_{26}A_{18}A_{20} + A_{27}A_{18}A_{22} - A_{25}A_{19}A_{22} - A_{27}A_{20}\ A_{24}\right),$

$B_3 = A_{20}\left({A_{16}}^2 - A_{17}A_{21}\right)\left(A_{26}\left({A_{18}}^2 - A_{19}A_{23}\right) - A_{27}A_{18}A_{24} + A_{25}A_{19}A_{24}\right).$

Appendix B

Coefficients 2

$$
\begin{aligned}
v_{1n} &= \frac{A_1^2A_4A_5A_9 - A_2A_4A_5A_6A_9 + A_2A_3A_7\xi_n^2 + A_1A_4A_7\xi_n^2 + A_2A_5A_9\xi_n^2}{A_5\Delta_n},\\
v_{2n} &= \frac{\xi_n(-A_2A_3^2A_7 + A_2A_4A_7A_8 - A_2A_3A_5A_9 - A_1A_4A_5A_9 - A_4A_7\xi_n^2)}{A_5\Delta_n},\\
v_{3n} &= 1.0\\
v_{4n} &= \frac{-A_1^2A_3A_5A_9 + A_2A_3A_5A_6A_9 - A_1A_3A_7\xi_n^2 - A_2A_7A_8\xi_n^2 + A_1A_5A_9\xi_n^2 + A_7\xi_n^4}{A_5\Delta_n},\\
v_{5n} &= \frac{\xi_n(-A_1A_3^2A_7 + A_1A_4A_7A_8 - A_1A_3A_5A_9 - A_4A_5A_6A_9 + A_3A_7\xi_n^2 + A_5A_9\xi_n^2)}{A_5\Delta_n},\\
v_{6n} &= \frac{-\xi_n}{A_5},\\
\Delta_n &= A_1^2A_3^2 - A_2A_3^2A_6 - A_1^2A_4A_8 + A_2A_4A_6A_8 - 2A_1A_3\xi_n^2 - A_4A_6\xi_n^2 - A_2A_8\xi_n^2 + \xi_n^4.
\end{aligned}
$$

Appendix C

Coefficients 3

$$
\begin{aligned}
V_{1n} &= \frac{A_{16}^2A_{19}A_{20}A_{24} - A_{17}A_{19}A_{20}A_{21}A_{24} + A_{17}A_{18}A_{22}\zeta_n^2 + A_{16}A_{19}A_{22}\zeta_n^2 + A_{17}A_{20}A_{24}\zeta_n^2}{A_{20}\Pi_n},\\
V_{2n} &= \frac{\zeta_n(-A_{17}A_{18}^2A_{22} + A_{17}A_{19}A_{22}A_{23} - A_{17}A_{18}A_{20}A_{24} - A_{16}A_{19}A_{20}A_{24} - A_{19}A_{22}\zeta_n^2)}{A_{20}\Pi_n},\\
V_{3n} &= 1.0\\
V_{4n} &= \frac{-A_{16}^2A_{18}A_{20}A_{24} + A_{17}A_{18}A_{20}A_{21}A_{24} - A_{16}A_{18}A_{22}\zeta_n^2 - A_{17}A_{22}A_{23}\zeta_n^2 + A_{16}A_{20}A_{24}\zeta_n^2 + A_{22}\xi_n^4}{A_{20}\Pi_n},\\
V_{5n} &= \frac{\zeta_n(-A_{16}A_{18}^2A_{22} + A_{16}A_{19}A_{22}A_{23} - A_{16}A_{18}A_{20}A_{24} - A_{19}A_{20}A_{21}A_{24} + A_{18}A_{22}\zeta_n^2 + A_{20}A_{24}\zeta_n^2)}{A_{20}\Pi_n},\\
V_{6n} &= \frac{-\zeta_n}{A_{20}},\\
\Pi_n &= A_{16}^2A_{18}^2 - A_{17}A_{18}^2A_{21} - A_{16}^2A_{19}A_{23} + A_{17}A_{19}A_{21}A_{23} - 2A_{16}A_{18}\zeta_n^2 - A_{19}A_{21}\zeta_n^2 - A_{17}A_{23}\zeta_n^2 + \zeta_n^4.
\end{aligned}
$$
